# Fuzzy rank functions in the set of all binary systems

**DOI:** 10.1186/s40064-016-3536-z

**Published:** 2016-10-24

**Authors:** Hee Sik Kim, J. Neggers, Keum Sook So

**Affiliations:** 1 Department of Mathematics, Hanyang University, Seoul, 04763 Korea; 2 Department of Mathematics, University of Alabama, Tuscaloosa, AL 35487-0350 USA; 3 Department of Mathematics, Hallym University, Chuncheon, 24252 Korea

**Keywords:** (Fuzzy) rank-subalgebra, (Fuzzy) rank-*d*-function, (Fuzzy) symmetric-rank-function, Right parallel, $\rho$-Shrinking, Selective, *Bin*(*X*), Primary 08A72, 20N02

## Abstract

In this paper, we introduce fuzzy rank functions for groupoids, and we investigate their roles in the semigroup of binary systems by using the notions of right parallelisms and $\rho$-shrinking groupoids.

## Background

The notion of a fuzzy subset of a set was introduced by Zadeh (1965). His seminal paper has opened up new insights and applications in a wide range of scientific fields. Rosenfeld (1971) used the notion of a fuzzy subset to set down corner stone papers in several areas of mathematics. Mordeson and Malik (1998) published a remarkable book, *Fuzzy commutative algebra*, presented a fuzzy ideal theory of commutative rings and applied the results to the solution of fuzzy intersection equations. The book included all the important work that has been done on *L*-subspaces of a vector space and on *L*-subfields of a field.

In the study of groupoids $(X,*)$ defined on set *X*, it has also proven useful to investigate the semigroups $(Bin(X), \Box )$ where *Bin*(*X*) is the set of all binary systems (groupoids) $(X,*)$ along with an associative product operation $(X,*)\,\Box \,(X, \bullet ) = (X, \Box )$ such that $x\,\Box \,y = (x*y)\,\bullet \,(y*x)$ for all $x, y\in X$. Thus, e.g., it becomes possible to recognize that the left-zero-semigroup $(X,*)$ with $x*y = x$ for all $x, y\in X$ acts as the identity of this semigroup [see Kim and Neggers (2008)]. Fayoumi (2011) introduced the notion of the center *ZBin*(*X*) in the semigroup *Bin*(*X*) of all binary systems on a set *X*, and showed that a groupoid $(X,\bullet )\in ZBin(X)$ if and only if it is a locally-zero groupoid. Han et al. (2012) introduced the notion of hypergroupoids $(HBin(X),\Box )$, and showed that $(HBin(X), \Box )$ is a supersemigroup of the semigroup $(Bin(X), \Box )$ via the identification $x \longleftrightarrow \{x\}$. They proved that $(HBin^*(X), \ominus , [\emptyset ])$ is a *BCK*-algebra.

In this paper, we introduce fuzzy rank functions for groupoids, and we investigate their roles in the semigroup of binary systems by using the notions of right parallelisms and $\rho$-shrinking groupoids.

## Preliminaries

Given a non-empty set *X*, we let $Bin(X)$ denote the collection of all groupoids $(X,*)$, where $*: X\times X\rightarrow X$ is a map and where $*(x, y)$ is written in the usual product form. Given elements $(X,*)$ and $(X,\bullet )$ of $Bin(X)$, define a product “$\Box$” on these groupoids as follows:

$$(X,*) \,\Box \,(X,\bullet ) =(X,\Box )$$

where

$$x\,\Box \,y = (x*y)\,\bullet \, (y*x)$$

for any $x, y\in X$. Using that notion, H. S. Kim and J. Neggers proved the following theorem.

### **Theorem 1**

(Kim and Neggers 2008) $(Bin(X), \Box )$ *is a semigroup, i.e., the operation* “$\Box$” *as defined in general is associative. Furthermore, the left- zero-semigroup is the identity for this operation*.

## Fuzzy rank functions for groupoids

Given a groupoid $(X,*)$ in *Bin*(*X*), a map $\rho : X\rightarrow [0, \infty )$ (or $\rho : X\rightarrow [0, 1]$) is said to be:

(i) a (*fuzzy*) *rank-subalgebra*: $\rho (x*y)\ge \min \{\rho (x), \rho (y)\}$,
(ii) a (*fuzzy*) *rank-co-subalgebra*: $\rho (x*y) \le \max \{\rho (x), \rho (y)\}$,
(iii) a (*fuzzy*) *rank-d-function*: $\rho (x*y) =\max \{ \rho (x) -\rho (y), 0\}$,
(iv) a (*fuzzy*) *symmetric-rank-function*: $\rho (x*y)= \max \{\rho (x)-\rho (y), \rho (y) -\rho (x)\}\,\hbox{for\,all}\,x,y \in X.$

Note that if $\rho : X\rightarrow [0, \infty )$ is a fuzzy subset of *X*, then it is a fuzzy rank-subalgebra as well. Thus these algebraic structures are special cases of the classes of fuzzy rank-subalgebras. Each of the types listed above serve as some idea of measure of size when the binary operation “$*$” is considered as corresponding very roughly to a (group) sum (product), a left-zero-semigroup, a *d*-algebra (*BCK*-algebra) or an absolute (value) difference. For the sake of being able to make comparisons in the behavior of interactions of rank-type and groupoid it seems a better idea to consider these simultaneously rather than study only isolated cases without considering common aspects as well as distinguishing ones.

### *Example 2*

- Let $(X,*)$ be a left-zero-semigroup, i.e., $x*y = x$ for all $x, y\in X$. If $\rho : X\rightarrow [0, \infty )$ is any map, then $\rho (x*y) = \rho (x)$ for all $x, y\in X$. It follows that $\rho (x*y) \ge \min \{\rho (x), \rho (y)\}$ and $\rho (x*y) \le \max \{\rho (x), \rho (y)\}$ for all $x, y\in X$, which shows that every function $\rho : X\rightarrow [0, \infty )$ is both a (fuzzy) rank-subalgebra and a (fuzzy) rank-co-subalgebra.
- Let $(X,*)$ be a left-zero-semigroup and let $\rho : X \rightarrow [0, \infty )$ be a (fuzzy) rank-*d*-function. Then it is a zero function. In fact, if we assume that there exists an $x_0\in X$ such that $\rho (x_0)> 0$, then $0< \rho (x_0) = \rho (x_0* y) =\max \{\rho (x_0)-\rho (y), 0\}$ for all $y\in X$. If we take $y:=x_0$, then it leads to a contradiction.
- Let $(X,*)$ be a left-zero-semigroup and let $\rho : X \rightarrow [0, \infty )$ be a (fuzzy) symmetric-rank-function. Assume that there exists $x_0\in X$ such that $0< \rho (x_0)$. Since $\rho$ is a (fuzzy) symmetric-rank-function, we have $\rho (x) = \rho (x*y) = \max \{\rho (x) -\rho (y), \rho (y)-\rho (x)\}$ for all $x, y\in X$. If we take $x:= x_0, y:= y_0$, then $0< \rho (x_0)= \rho (x_0* x_0) =\max \{ \rho (x_0)-\rho (x_0), \rho (x_0)-\rho (x_0)\}=0$, a contradiction. This shows that $\rho$ is a zero function.

If $(X,*)$ is a right-zero-semigroup, i.e., $x*y = y$ for all $x, y\in X$, then any function $\rho : X\rightarrow [0, \infty )$ is both a (fuzzy) rank-subalgebra and a (fuzzy) rank-co-subalgebra of $(X,*)$, while if $\rho$ is either a (fuzzy) rank-*d*-function or a (fuzzy) symmetric-rank-function, then it is a zero function.

A groupoid $(X,*)$ is said to be *selective* (Neggers 1976; Neggers and Kim 1996) if $x*y\in \{x, y\}$ for all $x, y\in X$. For example, every left-(right-)zero-semigroup is selective. Given a selective groupoid $(X,*)$, we may construct a digraph via $x\rightarrow y \Leftrightarrow x*y =y$ for all $x, y\in X$. Hence selective groupoids are interpretable as digraphs on the (vertex) set *X*.

### **Proposition 3**

*Let* $(X,*)$ *be a selective groupoid and let* $\rho : X\rightarrow [0, \infty )$ *be a* (*fuzzy*) *rank*-*d*-*function of* *X*. *If* $x\in X$ *such that* $\rho (x)>0$, *then any vertex* $y (\not = x)$ *in* *X* *with* $x\nrightarrow y$ *has* $\rho (y)=0$.

### *Proof*

Let $y (\not = x)$ in *X* with $x\nrightarrow y$. Since $(X,*)$ is selective and $x\nrightarrow y$, we have $x*y = x$. It follows from the fact that $\rho$ is a rank-*d*-function that $\rho (x) = \rho (x*y) = \max \{\rho (x)-\rho (y), 0\}$ and hence that $\rho (x) = \rho (x) -\rho (y)$, proving that $\rho (y)= 0$. $\square$

### **Theorem 4**

*Let* $(X,*)$ *be a selective groupoid and let* $\rho : X\rightarrow [0, \infty )$ *be a* (*fuzzy*) *rank*-*d*-*function of* *X*. *If* $x\in X$ *such that* $\rho (x)>0$, *then there exists at most one vertex* $x\in X$ *such that* $\rho (x)>0$.

### *Proof*

Assume that there are two vertices *x* and *y* in *X* such that $\rho (x)>0, \rho (y)>0$. It follows that $x\rightarrow y, y\rightarrow x$ by Proposition 3. Now $x\rightarrow y$ implies that $x*y= y$. Hence $0 < \rho (y) =\rho (x*y) =\max \{\rho (x)-\rho (y), 0\}= \rho (x)-\rho (y)$. This shows that $2\rho (y) = \rho (x)$. Similarly, $y\rightarrow x$ implies $2\rho (x) = \rho (y)$. Thus we obtain $\rho (x) = 2\rho (y) = 4\rho (x)$, which implies $\rho (x)=0$, a contradiction. $\square$

### **Proposition 5**

*If* $(X,*)$ *is a selective groupoid, then every* (*fuzzy*) *rank*-*d*-*function of* $(X, *)$ *is a zero function*.

### *Proof*

If $(X,*)$ is a selective groupoid, then $x*x = x$ for all $x\in X$. Since $\rho$ is a (fuzzy) rank-*d*-function, we obtain $\rho (x) = \rho (x*x) = \max \{\rho (x)-\rho (x), 0\} = 0$, proving the proposition. $\square$

Given a map $\rho : X\rightarrow [0, \infty )$, we define

$${\sum _i(\rho )}:= \left\{ (X,*)\in Bin(X)| \text {the\,condition}\, (i) \text {holds\,for}\, (X,*)\right\} \quad (i=1, 2, 3, 4).$$

### **Proposition 6**

$(\sum _i(\rho ), \Box )$ *is a subsemigroup of* $(Bin(X), \Box )$ *where* $i= 1, 2, 3$.

### *Proof*

Let $(X,*), (X,\bullet )\in \sum _1(\rho )$. Then $\rho (x*y)\ge \min \{\rho (x), \rho (y)\}$ and $\rho (x\bullet y)\ge \min \{\rho (x), \rho (y)\}$ for all $x, y\in X$. If we let $(X,\Box ):= (X,*)\,\Box \,(X,\bullet )$, then for any $x, y\in X$, we have $x\,\Box \,y = (x*y)\bullet (y*x)$. It follows that

$$\begin{aligned} \rho (x\,\Box \,y)&= {} \rho ((x*y)\,\bullet \, (y*x))\\&\ge {} \min \{\rho (x*y), \rho (y*x)\}\\&\ge {} \min \{ \min \{\rho (x), \rho (y)\}, \min \{\rho (y), \rho (x)\}\}\\&= {} \min \{ \rho (x), \rho (y)\} \end{aligned}$$

This shows that $(X,*)\,\Box \,(X,\bullet )= (X, \Box )\in \sum \nolimits _1(\rho )$. Hence $(\sum \nolimits _1(\rho ), \Box )$ is a subsemigroup of $(Bin(X), \Box )$. Similarly, we may obtain that $(\sum \nolimits _i(\rho ), \Box )$ is also a subsemigroup of $(Bin(X), \Box )$ where $i= 2, 3$. $\square$

Note that $(\sum \nolimits _4(\rho ), \Box )$ need not be a subsemigroup of $(Bin(X), \Box )$. Let $(X,*), (X,\bullet )\in \sum \nolimits _1(\rho )$ and let $(X,\Box ):= (X,*)\,\Box \,(X,\bullet )$. If we take $x, y\in X$ such that $\rho (x) > \rho (y)$, then we have

$$\begin{aligned} \rho (x\,\Box \,y)&= {} \rho ((x*y)\,\bullet \, (y*x))\\&= {} \max \{\rho (x*y)-\rho (y*x), \rho (y*x)-\rho (y*x)\}\\&= {} \max \{ \max \{\rho (x)-\rho (y), \rho (y)-\rho (x)\}\\&\quad- \max \{\rho (y)-\rho (x), \rho (x)-\rho (y)\},\\&\quad \max \{\rho (y)-\rho (x), \rho (x)-\rho (y)\} \\&\quad-\max \{\rho (x)-\rho (y), \rho (y)-\rho (x)\} \}\\&= {} \min \left\{ [\rho (x)- \rho (y)] - [\rho (x) -\rho (y)],\right. \\&\left. \quad [\rho (x)- \rho (y)] - [\rho (x) -\rho (y)]\right\} \\&= {} 0 < \rho (x) -\rho (y) \\&= {} \max \{ \rho (x) -\rho (y), \rho (y) -\rho (x)\}, \end{aligned}$$

which shows that $(\sum \nolimits _4(\rho ), \Box )$ is not a subsemigroup of $(Bin(X), \Box )$.

Let $\rho : X\rightarrow [0, \infty )$ be a map. A groupoid $(X,*)$ is said to have a $\rho$-*chain* *n* if there exist $x_1, \ldots , x_n\in X$ such that $\rho (x_1)< \rho (x_2)< \cdots < \rho (x_n)$. We denote the $\rho$-chain by $\langle x_1, \ldots , x_n \rangle$. A groupoid $(X,*)$ is said to have the $\rho$-*height* *n* if $\langle x_1, \ldots , x_n \rangle$ is the largest maximal $\rho$-chain in $(X,*)$.

### **Proposition 7**

*Let* $\rho : X\rightarrow [0, \infty )$ *be a map and let* $Bin(X) = \sum _1(\rho )$. *Then the* $\rho$-*height of* $(X,*)$ *is* ≥2 *for any* $(X,*)\in Bin(X)$.

### *Proof*

Assume there exist $x, y, z\in X$ such that $\rho (x)< \rho (y) < \rho (z)$. Let $(X,*)$ be a groupoid such that $x= y*z$. Then $(X,*) \in Bin(X) = \sum \nolimits _1(\rho )$. It follows that $\rho (y*z) =\rho (x) < \rho (y) = \min \{\rho (y), \rho (z)\}$. This shows that $(X,*)\not \in \sum \nolimits _1(\rho ) = Bin(X)$, a contradiction. $\square$

### **Proposition 8**

*Let* $\rho : X\rightarrow [0, \infty )$ *be a map and let* $Bin(X) = \sum _1(\rho )$. *If* $\rho$ *has two values* $a, b\in [0, \infty )$ *with* $a< b$, *then there exists uniquely an* $\widehat{x} \in X$ *such that* $\rho (\widehat{x})= b$ *and* $\rho (y) = a$ *for all* $y\not = \widehat{x}$ *in* *X*.

### *Proof*

Let $\widehat{x} \in X$ such that $\rho (\widehat{x}) = b > a$. If $y\in X$ such that $\widehat{x}\not = y$, then $\rho (y) = a$. In fact, if $\rho (y)=b$, then we may take a groupoid $(X,*)$ in *Bin*(*X*) such that $\rho (\widehat{x} * y) = a$, since $Bin(X) = \sum\nolimits _1(\rho )$. It follows that $a = \rho (\widehat{x} * y) \ge \min \{ \rho (\widehat{x}), \rho (y)\}= b$, a contradiction. We claim that such an $\widehat{x}$ is unique. Assume that there are two elements $x^{\prime }, \widehat{x}$ in *X* such that $\rho (\widehat{x}) = b = \rho (x^{\prime })$. A groupoid $(X,\bullet )$ satisfying $\rho (\widehat{x}\,\bullet \, x^{\prime }) = a$ may then be selected. It follows that $a= \rho (\widehat{x}\,\bullet \, x^{\prime }) \ge \min \{ \rho (\widehat{x}), \rho (x^{\prime })\}= b$, a contradiction. $\square$

### **Proposition 9**

*Let* $(X,*)\in Bin(X)$ *and let* $\rho : X\rightarrow [0, \infty )$ *be a* (*fuzzy*) *rank*-*d*-*function of* *X*. *Then*

1. $$Ker(\rho ) \not = \emptyset$$,
2. *if* $$\rho (x) \le \rho (y)$$ *then* $$x*y \in Ker(\rho )$$,
3. $Ker(\rho )$ *is a right ideal of* $(X,*)$.

### *Proof*

(1) Given $x\in X$, $\rho (x*x) = \max \{\rho (x)-\rho (x), 0\} = 0$. It follows that $x*x\in Ker(\rho )$. (2) If $\rho (x) \le \rho (y)$ then $\rho (x*y) = \max \{\rho (x)-\rho (y), 0\}= 0$ and hence $x*y\in Ker(\rho )$. (3) Given $x\in Ker(\rho ), y\in X$, we have $\rho (x*y) = \max \{\rho (x)-\rho (y), 0\} = \max \{0-\rho (y), 0\} = 0$, which shows $x*y\in Ker(\rho )$. $\square$

## Right parallelisms

Let $\rho _1, \rho _2$ be mappings from *X* to $[0, \infty )$. The map $\rho _1$ is said to be *right parallel* to $\rho _2$ if $\rho _1(a)\le \rho _1(b)$ implies $\rho _2(a)\le \rho _2(b)$, and we denote it by $\rho _1\, ||\, \rho _2$.

### **Proposition 10**

*If* $\rho _1$ *is a* (*fuzzy*) *rank-subalgebra of* $(X,*)$ *and* $\rho _1 || \rho _2$, *then* $\rho _2$ *is also a* (*fuzzy*) *rank-subalgebra of* $(X,*)$.

### *Proof*

If $\rho _1$ is a rank-subalgebra of $(X,*)$, then $\rho _1(x*y)\ge \min \{\rho _1(x),$ $\rho _1(y)\}$ for all $x, y\in X$. Without loss of generality, we let $\rho _1(x*y) \ge \rho _1(x)$. Since $\rho _1|| \rho _2$, we obtain $\rho _2(x*y) \ge \rho _2(x)$. It follows that $\rho _2(x*y)\ge \min \{\rho _2(x), \rho _2(y)\}$. $\square$

Given maps $\rho _i: X\rightarrow [0, \infty )$, we define

$$\begin{aligned} (\rho _1 + \rho _2)(x):&= {} \rho _1(x) + \rho _2(x)\\ (\rho _1 \,\bullet \, \rho _2)(x):&= {} \rho _1(x)\rho _2(x) \end{aligned}$$

for all $$x\in X$$.

Note that $\rho _1 + \rho _2$ need not be a (fuzzy) rank-subalgebra of $(X,*)$ even though $\rho _1$ and $\rho _2$ are (fuzzy) rank-subalgebras of $(X,*)$. In fact,

$$\begin{aligned} (\rho _1 + \rho _2)(x*y)&= {} \rho _1(x*y) + \rho _2(x*y)\\&\ge {} \min \{\rho _1(x), \rho _1(y)\} + \min \{\rho _2(x), \rho _2(y)\} \end{aligned}$$

and

$$\min \{ (\rho _1 + \rho _2)(x), (\rho _1 + \rho _2)(y)\} = \min \{\rho _1(x) + \rho _2(x), \rho _1(y) + \rho _2(y)\}$$

In the real numbers, it is not always true that $\min \{a, b\} + \min \{c, d\} \ge \min \{a+c, b+d\}$, which shows that $\rho _1 + \rho _2$ need not be a (fuzzy) rank-subalgebra of $(X,*)$.

### **Proposition 11**

*Let* $\rho _1, \rho _2$ *be mappings from* *X* *to* $[0, \infty )$. *If* $\rho _1$ *is a constant for all* $x\in X$ *and if* $\rho _1|| \rho _2$, *then* $\rho _2$ *is also a constant function on* *X*.

### *Proof*

Straightforward. $$\square$$

Note that any map $\rho _1$ is right parallel to $\rho _2$ if $\rho _2$ is a constant function. The ‘right parallel’ relation “||” is reflexive and transitive, but it is not an anti-symmetric, i.e., || is a quasi order, but not a partial order on $\{\rho | \rho : X\rightarrow [0, \infty )$ : a map $\}$.

For example, we let $X:=[0, \infty )$ and let $\rho _1: X\rightarrow [0, \infty )$ be the identity map, and let $\rho _2: X\rightarrow [0, \infty )$ be a map defined by $\rho _2(x):= e^ x$. Then $\rho _1(a)\le \rho _1(b)$ if and only if $\rho _2(a)\le \rho _2(b)$ for all $a, b\in X$, i.e., $\rho _1|| \rho _2, \rho _2|| \rho _1$, but $\rho _1 \not = \rho _2$.

### **Proposition 12**

*If* $\rho _1$ *is a* (*fuzzy*) *rank-subalgebra of* $(X,*)$ *and* $\rho _1|| \rho _2$, *then* $\rho _1\,\bullet \,\rho _2$ *is also a* (*fuzzy*) *rank-subalgebra of* $(X,*)$.

### *Proof*

If $\rho _1$ is a rank-subalgebra of $(X,*)$, then $\rho _1(x*y)\ge \min \{\rho _1(x),$ $\rho _1(y)\}$ for all $x, y\in X$. If we assume $\rho _1(x*y)\ge \rho _1(x)$, then

$$\begin{aligned} (\rho _1\,\bullet \,\rho _2)(x*y)&= {} \rho _1(x*y) \rho _2(x*y)\\&\ge {} \rho _1(x) \rho _1(y)\\&= {} (\rho _1\,\bullet \,\rho _2)(x)\\&\ge {} \min \{(\rho _1\,\bullet \,\rho _2)(x), (\rho _1\,\bullet \,\rho _2)(x)\}, \end{aligned}$$

which proves the proposition. $\square$

### **Corollary 13**

*If* $\rho _1$ *is a* (*fuzzy*) *rank-co-subalgebra of* $(X,*)$ *and* $\rho _1|| \rho _2$, *then* $\rho _1\,\bullet \,\rho _2$ *and* $\rho _2$ *are also* (*fuzzy*) *rank-co-subalgebras of* $(X,*)$.

### *Proof*

The proofs are similar to Propositions 10 and 12. $\square$

In the above note, we mentioned that $\rho _1 + \rho _2$ need not be a rank-subalgebra of $(X,*)$. Using the notion of the right parallelism, we prove the following.

### **Proposition 14**

*If* $\rho _i$ *is a rank-subalgebra of* $(X,*)$ $(i= 1,2)$ *and if* $\rho _1|| \rho _2$, *then* $\rho _1 + \rho _2$ *is also a rank-subalgebra of* $(X,*)$.

### *Proof*

Since $\rho _1$ is a rank-subalgebra of $(X,*)$, we have $\rho _1(x*y)\ge \min \{\rho _1(x), \rho _1(y)\}$ for all $x, y\in X$. Without loss of generality, we let $\rho _1(x*y)\ge \rho _1(x)$. Since $\rho _1|| \rho _2$, we obtain $\rho _2(x*y)\ge \rho _2(x)$. It follows that $\rho _1(x*y) + \rho _2(x*y) \ge \rho _1(x) + \rho _2(x)$ and hence $(\rho _1 + \rho _2)(x*y) \ge \min \{ (\rho _1 + \rho _2)(x), (\rho _1 + \rho _2)(y)\}$, proving the proposition. $\square$

### **Corollary 15**

*If* $\rho _i$ *is a rank-co-subalgebra of* $(X,*)$ $(i= 1,2)$ *and if* $\rho _1|| \rho _2$, *then* $\rho _1 + \rho _2$ *is also a rank-co-subalgebra of* $(X,*)$.

### *Proof*

Similar to Proposition 14. $\square$

### **Proposition 16**

*Let* $X:={\mathbf{R}}$ *be the set of all real numbers. Let* $\rho _1, \rho _2$ *be mappings from* *X* *to* $[0, \infty )$ *and let* $\rho _2(x):=x$ *for all* $x\in X$. *If* $\rho _1|| \rho _2$, *then* $\rho _1$ *is strictly increasing.*

### *Proof*

Assume that there are $x, y\in X$ such that $x<y, \rho _1(x) \ge \rho _1(y)$. Since $\rho _1|| \rho _2$, we obtain $x=\rho _2(x)\ge \rho _2(y) = y$, a contradiction. $\square$

### **Theorem 17**

*If* $\rho _i$ *is a rank*-*d*-*function of* $(X,*)$ $(i= 1,2)$ *and if* $\rho _1|| \rho _2$, *then* $\rho _1 + \rho _2$ *is also a rank*-*d*-*function of* $(X,*)$.

### *Proof*

Given $x, y\in X$, we have four cases: (i) $\rho _1(x) \ge \rho _1(y), \rho _2(x) \ge \rho _2(y)$; (ii) $\rho _1(x) \ge \rho _1(y), \rho _2(x) < \rho _2(y)$; (iii) $\rho _1(x) < \rho _1(y), \rho _2(x) \ge \rho _2(y)$; (iv) $\rho _1(x)< \rho _1(y), \rho _2(x) < \rho _2(y)$. Since $\rho _1|| \rho _2$, the cases (ii) and (iii) are removed. For the case (i), we have $\rho _1(x*y) =\rho _1(x) -\rho _1(y)$ and $\rho _2(x*y)=\rho _2(x) -\rho _2(y)$. It follows that

$$\begin{aligned} (\rho _1+ \rho _2)(x*y)&= {} \rho _1(x*y) + \rho _2(x*y)\\&= {} \rho _1(x) - \rho _1(y) + \rho _2(x) - \rho _2(y)\\&= {} (\rho _1 +\rho _2)(x)- (\rho _1 +\rho _2)(y)\\&\le {} \max \{(\rho _1+\rho _2)(x)-(\rho _1+\rho _2)(y), 0\}, \end{aligned}$$

For the case (iv), we have $\rho _1(x*y) = 0 = \rho _2(x*y)$. It follows that $(\rho _1+ \rho _2)(x*y) = \rho _1(x*y) + \rho _2(x*y)=0 =\max \{(\rho _1+\rho _2)(x)-(\rho _1+\rho _2)(y), 0\}$, proving the theorem. $\square$

Note that if $\rho _1|| \rho _2, \rho _2 || \rho _3$, then $\rho _1 || \rho _2 + \rho _3$, and if $\rho _1 || \rho _2, \rho _1 || \rho _3$, then $\rho _1 || \rho _2 + \rho _3$.

## $\rho$-Shrinking groupoids

A groupoid $(X,*)$ is said to be $\rho$-*shrinking* if $\rho : X\rightarrow [0, \infty )$ is a map satisfying the condition:

$$\rho (x*y) \le \min \{\rho (x), \rho (y)\}$$

for all $$x, y\in X$$.

### *Example 18*

Let $X:=[0, \infty )$ and let $x*y:= x +y$ for all $x, y\in X$. If we define $\rho (x):=x$ for all $x\in X$, then $\rho (x*y)\ge \max \{\rho (x), \rho (y)\}$. It follows that $(e^{-\rho })(x*y) = e^{-x}e^{-y}\le \min \{e^{-x}, e^{-y}\}= \min \{ (e^{-\rho })(x),$ $(e^{-\rho })(y)\}$, which shows that $(X,*)$ is $e^{-\rho }$-shrinking.

### **Theorem 19**

*If* $(X,*)$ *is both* $\rho _1$-*shrinking and* $\rho _2$-*shrinking and if* $\rho _1|| \rho _2$, *then* $(X,*)$ *is both* $(\rho _1 + \rho _2)$-*shrinking and* $\rho _1\,\bullet \,\rho _2$-*shrinking.*

### *Proof*

Since $(X,*)$ is $\rho _1$-shrinking, we have $\rho _1(x*y) \le \min \{\rho _1(x),$ $\rho _1(y)\}$ for all $x, y\in X$. The condition $\rho _1 || \rho _2$ implies that $\rho _2(x*y) \le \min \{\rho _2(x), \rho _2(y)\}$. Hence

$$\begin{aligned} (\rho _1+ \rho _2)(x*y)&= {} \rho _1(x*y) + \rho _2(x*y)\\&\le {} \min \{\rho _1(x), \rho _1(y)\} + \min \{\rho _2(x), \rho _2(y)\} \end{aligned}$$

Without loss of generality, we may assume $\rho _1(x)\le \rho _1(y)$. Then $\rho _2(x)\le \rho _2(y)$, since $\rho _1 || \rho _2$. It follows that $(\rho _1+ \rho _2)(x*y) \le \rho _1(x) + \rho _2(y) = (\rho _1+ \rho _2)(x)$. Hence $(X,*)$ is $(\rho _1 + \rho _2)$-shrinking. Similarly, if we assume $\rho _1(x)\le \rho _1(y)$, then

$$\begin{aligned} (\rho _1\,\bullet \, \rho _2)(x*y)&= {} \rho _1(x*y)\rho _2(x*y)\\&\le {} \rho _1(x)\rho _1(x)\\&= {} (\rho _1\,\bullet \,\rho _2)(x)\\&= {} \min \{(\rho _1\,\bullet \,\rho _2)(x), (\rho _1\,\bullet \,\rho _2)(y)\}, \end{aligned}$$

which shows that $(X,*)$ is $\rho _1\,\bullet \, \rho _2$-shrinking. $\square$

### **Proposition 20**

*If* $(X,*)$ *and* $(X,\bullet )$ *are* $\rho$-*shrinking and if* $(X,\Box ):=(X,*)\,\Box \,(X,\bullet )$, *then* $(X,\Box )$ *is also* $\rho$-*shrinking*.

### *Proof*

If $(X,\Box ):=(X,*)\,\Box \,(X,\bullet )$, then for all $x, y\in X$, we have

$$\begin{aligned} \rho (x\,\Box \,y)&= {} \rho ((x*y)\,\bullet \,(y*x))\\&\le {} \min \{\rho (x*y), \rho (y*x)\}\\&\le {} \min \{\min \{\rho (x), \rho (y)\}, \min \{\rho (y), \rho (x)\} \\&= {} \min \{\rho (x), \rho (y)\}, \end{aligned}$$

showing that $(X,\Box )$ is $\rho$-shrinking. $\square$

Proposition 20 shows that the collection of all $\rho$-shrinking groupoids forms a subsemigroup of $(Bin(X), \Box )$.

Given maps $\rho : X\rightarrow [0, \infty )$ and $\sigma : Y\rightarrow [0, \infty )$, we define a map $[\rho , \sigma ]: X\times Y\rightarrow [0, \infty )$ by $[\rho , \sigma ](x):= \rho (x) + \sigma (y)$ as a sort of “inner product” ranking. Given groupoids $(X,*)$ and $(Y,\bullet )$, we define a Cartesian product $(X\times Y, \nabla )$ where $(x, y)\nabla (x^{\prime }, y^{\prime }):= (x*x^{\prime }, y\,\bullet \, y^{\prime })$ for all $(x, y), (x^{\prime }, y^{\prime })\in X\times Y$.

### **Proposition 21**

*If* $(X,*)$ *is* $\rho$-*shrinking and* $(Y,\bullet )$ *is* $\sigma$-*shrinking, then* $(X\times Y, \nabla )$ *is* $[\rho , \sigma ]$-*shrinking*.

### *Proof*

Since $(X,*)$ is $\rho$-shrinking and $(Y,\bullet )$ is $\sigma$-shrinking, we have $\rho (x*y)\le \min \{\rho (x), \rho (y)\}$ and $\sigma (x\,\bullet \, y) \le \min \{\sigma (x), \sigma (y)\}$ for all $x, y\in X$. It follows that

$$\begin{aligned} \rho (x*y) + \sigma (x\,\bullet \, y)&\le {} \min \{\rho (x), \rho (y)\} + \min \{\sigma (x), \sigma (y)\}\\&\le {} \min \{\rho (x) + \sigma (x), \rho (y)+ \sigma (y)\}\\&= {} \min \{[\rho , \sigma ](x), [\rho ,\sigma ](y)\}, \end{aligned}$$

which proves the proposition. $\square$

## Conclusions

Above, we introduced four (fuzzy) rank functions in the semigroup of all binary systems (i.e., groupoids), and we investigated their roles related to selective groupoids and the notion of *Bin*(*X*). Using the notion of “right parallelism”, we showed that if $\rho _i$ is a (fuzzy) rank-subalgebra (resp., (fuzzy) rank-*d*-function) of $(X,*)$ ($i= 1,2$) and if $\rho _1|| \rho _2$, then $\rho _1 + \rho _2$ is also a (fuzzy) rank-subalgebra (resp., (fuzzy) rank-*d*-function) of $(X,*)$. By introducing the notion of $\rho$-shrinking to groupoids, we found that if $(X,*)$ is both $\rho _1$-shrinking and $\rho _2$-shrinking and if $\rho _1|| \rho _2$, then it is both $(\rho _1 + \rho _2)$-shrinking and $\rho _1\,\bullet \,\rho _2$-shrinking. This research may provide hyper-fuzzy rank functions in the set of all binary systems naturally, and thus several well-developed theorems/propositions in the areas of soft fuzzy theory and intuitionistic fuzzy set theory can then possibly be applied in future research also.
